# Historical root of precision medicine: an ancient concept concordant with the modern pharmacotherapy

**DOI:** 10.1186/s40199-017-0173-1

**Published:** 2017-04-04

**Authors:** Reihaneh Moeini, Zahra Memariani, Parvin Pasalar, Narjes Gorji

**Affiliations:** 1 Traditional Medicine and History of Medical Sciences Research Center, Department of Traditional Iranian Medicine, School of Traditional Iranian Medicine, Babol University of Medical Sciences, Babol, Iran; 2 Department of Clinical Biochemistry, Faculty of Medicine, Tehran University of Medical Sciences, Tehran, Iran

**Keywords:** Pharmacoproteomics, Personalized medicine, Avicenna, Persian medicine

## Abstract

Pharmacogenomics and pharmacoproteomics are new sciences that their goal is achieving therapeutics with maximum results and minimal side effects for each individual due to the pattern of his genome and proteome.

Although they considered new and high technology sciences but in distant past, Persian sages like Avicenna also knew about importance of “personalized medicine” and used specific patterns to detect individual differences in order to select suitable medication.

Based on experience and analogy they divided individuals into different categories considering characteristics like body color, body temperature, sleep-awake pattern and skeletal structure.

They also paid attention to effect of environmental conditions such as climate, job and the change of seasons on the influence of medication.

Considering the low cost and ease of use of these experiences, it seems that researching their opinions can uncover the historical roots of modern pharmacoproteomics and can possibly infuse new ideas in this field.

## Main text

During the past two decades "personalized medicine" and "precision medicine" have gained widespread attention. In which choosing the appropriate medication is based on the personal characteristics and individual differences [1]. Based on pharmacogenomics approaches, mapping the human genome in various diseases were applied to find the most effective medication with the least side effects for different people [1, 2]. Moreover most diseases are multi-factorial and caused by the interaction of genetic and environmental factors because the proteome as a dynamic entity specific to cells and tissues is affected by the environment; therefore multifactorial diseases could be redefined based on proteomics [2, 3].

Pharmacogenomics and pharmacoproteomics are new scientific fields and have an important role in the progression of personalized medicine in recent years. Surprisingly, the same concepts were cited by Avicenna-the most famous scientist in traditional Persian medicine (PM)-going back to as early as 1,000 years ago.

Avicenna (AD 980 to 1037) in the fourth section of first volume of his major encyclopedia “The Canon of Medicine” has expressed several interesting points about choosing and prescribing medication pertaining to personalized medicine. He explicitly said: “Any medication will have different effects on different bodies, organs of a person, at two different time points in one person's body, and in one organ at two different time points.”

He also described multiple factors that influence effectiveness of medications on human body. One of the most important of them being the temperament of person and target organ, and the others are gender, age, habits, season, climate, occupation, physical strength and physique [4].

As a fundamental concept in PM, temperament (*Mizaj*) is the summed attributes of the body (or any of its organs) and is related to its metabolism, behavior, and mental profile of the individuals; which roughly equals the term "phenotype" in modern medicine. Inherent temperament (*Mizaj e jebeli*) is expressed during the lifetime based on the genotype or could be affected by environmental conditions and lifestyle, resulting in acquired temperament (*Mizaj e arezi*) [4–6]. Based on Avicenna's theory, both of these temperaments are important in medication selection [4].

Ten major factors were examined by Avicenna and his followers to determine a person's temperament, including color of face and body, skeletal structure, hair features, sleep-wake pattern, body excretions feces, urine and sweat, behavior, mental states and mood [4–6].

Considering these characteristics, temperaments are basically determined in different spectrums of four basic qualities: coldness or hotness, dryness or wetness. In their approach, inherent and acquired temperaments were of importance in targeted therapy as markers of genotype, and they believed that environmental factors could change temperamental features and thus the effectiveness of treatment [4–6].

As such, they believed that effectiveness of many drugs with the same dose is different in hot and cold temperaments, such as scammony resin (*Convolvulus scammonia* L*.*) which will be effective in people with hot temperament while has no effect in people with cold temperament. Also nutritional habits can affect the body’s response to the new drugs; and people with different diets need different treatments and medicines [5].

Matching today’s pharmacokinetics’ viewpoints, they also stated that some medications will be less effective or have paradoxical response in the elderly compared to young patients, Rose (*Rosa domascena* L*.*) infused oil which will have cooling effects in the elderly compared to heating effects in the youth. They believed that the medicinal effects of one drug in one body could differ based on the season and climate because of environment’s temperature; so people who live in deserts need different treatments from mountaineers. For example, some toxins are not as lethal in cold regions as in the tropics [5]. Also, concerning the occupation they considered different treatments for patients with heavy physical work [3].

For these all situations, he anticipated a change in the drug dosage, alternative medications, or combining medications for optimizing the therapy. He also pointed the probability of drug tolerance and warned about the long term use of a medicine or therapy [4].

Avicenna conceptualized the influence of individual characteristics, life style and environment on pharmacotherapy which is in line with the modern concepts of effect of genetics and environment on pharmaco-metabolism. It is of note that he and his followers in Traditional Persian Medicine well understood these concepts by focusing on merely phenotypes [4].

This viewpoint (therapy selection based on personal characteristics) had been used as a principle of treatment by traditional Persian Physicians for a long time. Moreover, this method is inexpensive and more feasible for clinical assessment. They documented their clinical experience notes in their books which is an excellent material for future research and preliminary studies started for evaluation of conformity between these theories and modern knowledge [6]. As a result, it seems that researching their opinions can uncover the historical roots of modern pharmacoproteomics and can possibly infuse new ideas in this field.

## Abbreviations

PM: Persian medicine
